# Immunometabolic rewiring in tumorigenesis and anti-tumor immunotherapy

**DOI:** 10.1186/s12943-021-01486-5

**Published:** 2022-01-21

**Authors:** Xin Lian, Kailin Yang, Renliang Li, Maomao Li, Jing Zuo, Bohao Zheng, Wei Wang, Ping Wang, Shengtao Zhou

**Affiliations:** 1 Department of Obstetrics and Gynecology, Key Laboratory of Birth Defects and Related Diseases of Women and Children of MOE and State Key Laboratory of Biotherapy, West China Second Hospital, Sichuan University and Collaborative Innovation Center, Chengdu, 610041 Sichuan China; 2 Department of Radiation Oncology, Taussig Cancer Center, Cleveland Clinic, Cleveland, OH 44195 USA; 3 Department of Obstetrics and Gynecology, Wenzhou People’s Hospital, Wenzhou, Zhejiang, 325000 China; 4 West China School of Basic Medical Sciences & Forensic Medicine, Chengdu, 610041 Sichuan China; 5 Department of Pathology, West China Second Hospital, Sichuan University, Chengdu, 610041 Sichuan China

**Keywords:** Metabolic reprogramming, Oncometabolites, Immune cells, Tumor microenvironment, Immunotherapy

## Abstract

Cellular metabolism constitutes a fundamental process in biology. During tumor initiation and progression, each cellular component in the cancerous niche undergoes dramatic metabolic reprogramming, adapting to a challenging microenvironment of hypoxia, nutrient deprivation, and other stresses. While the metabolic hallmarks of cancer have been extensively studied, the metabolic states of the immune cells are less well elucidated. Here we review the metabolic disturbance and fitness of the immune system in the tumor microenvironment (TME), focusing on the impact of oncometabolites to the function of immune cells and the clinical significance of targeting metabolism in anti-tumor immunotherapy. Metabolic alterations in the immune system of TME offer novel therapeutic insight into cancer treatment.

## The basics of metabolic rewiring of immune cells in cancer biology

Cells uptake a variety of nutrients and undergo different biochemical processes to sustain growth and division. There are three major fundamental building blocks for cellular metabolism: glucose, fatty acid, and amino acid. In the neoplastic tissue, both cancer cells and stromal cells display metabolic characteristics of proliferating cells, implicating the metabolic similarities between them. For tumor cells, even in the presence of oxygen, glucose is catalyzed by a series of enzymes to generate lactate instead of being thoroughly oxidized into carbon dioxide. This phenomenon is called aerobic glycolysis, also known as Warburg effect. Apart from the glycolytic phenotype, alterations in lipid metabolism, including fatty acid synthesis, β-oxidation, and cellular lipid composition dramatically change in cancer cells as well. Amino acid metabolism is also frequently altered in cancer cells under the context of both tumorigenesis and cancer progression. These cell-intrinsic metabolic disturbances altogether contribute to a highly acidic, nutrient-deficient and hypoxic TME, which further aggravates the processes of metabolic reprogramming in both tumor cells and immunocytes of the tumor niche. For example, in the TME, glucose deprivation inhibits glycolysis in T cells, inducing anergy under which T cells fail to augment cytokines secretion and proliferation in response to stimulation [1]. Glucose deprivation also increases the ratio of AMP: ATP through activating AMP-activated protein kinase (AMPK) but inhibiting mammalian target of rapamycin (mTOR) and hypoxia-inducible factor 1α (HIF-1α), which supports differentiation of CD4^+^ T cells into immunosuppressive regulatory T cells (Tregs) rather than CD4^+^ effector T cells (Teffs) and promotes phenotype generation of anti-inflammatory M2 macrophages in excess of M1 macrophages [2–6]. In addition, accumulation of lactate in the TME inhibits proliferation and cytokine secretion of CD8^+^ T cells through impeding mitogen-activated protein kinase (MAPK) signaling and induces anergy of CD8^+^ T cells due to lactic acidosis [7–9]. Lactate is taken up by macrophages to induce differentiation into the immunosuppressive M2 macrophages through inducing arginase 1 (ARG1) expression [10]. For immunocytes, signaling programs can affect their immunological function through metabolism. For example, metabolic reprogramming in activated T cells is closely associated with two indispensable signals, antigen recognition through T-cell receptor (TCR) and co-stimulation [11]. When both are present, glucose transporters and enzymes associated with glycolysis become up-regulated and T cells are activated with proliferation and cytokines generation such as interleukin-2 (IL-2) [12, 13]. Glycolysis also regulates transcription and translation in T cells. For example, when restricting glucose consumption through inhibiting lactate dehydrogenase (LDH) in CD4^+^ T cells, reduced production of acetyl-coenzyme A (acetyl-CoA) results in deficient acetylation of histones at the locus of interferon-γ (IFN-γ) genes, and thus the production of IFN-γ is reduced [14]. In the hypoxia-driven hexosamine biosynthetic pathway (HBP), glycolysis intermediate fructose-6-phosphate (Fru6P) can be used to generate uridine diphosphate N-acetylglucosamine (UDP-GlcNAc) through a series of enzymes. UDP-GlcNAc is used for post-translational modifications such as O-GlcNAcylation of proteins important for differentiation and proliferation of immune cells [12]. In addition to glycolysis, lipid metabolism plays an important role in immune cells. Lipids are essential to cellular membrane biosynthesis, which is essential for proliferation. Acetyl-CoA carboxylase 1 (ACC1) is the rate-limiting enzyme of fatty acid biosynthesis. Deficiency of ACC1 inhibits the proliferation and survival of T cells in the antigen-specific response [12, 15]. Apart from above-mentioned fatty acid biosynthesis, fatty acid oxidation (FAO) can also affect immune cells. The rate-limiting enzyme of FAO, carnitine palmitoyltransferase 1A (CPT1A), is increased in memory CD8^+^ T cells, maintaining their long-term survival after eliminating antigens and generating rapid immune responses when rechallenged by antigen [16–18]. Cholesterol metabolism plays a positive role in T cell activation through maintaining fluid cell membrane which promotes TCR clustering [19]. Acyl-coenzyme A (Acyl-CoA) and free cholesterols are catalyzed by acyl-CoA:cholesterol transferase 1 (ACAT1) to generate cholesterol ester for storing free cholesterols. Inhibiting ACAT1 pharmacologically or genetically increases intracellular cholesterol level in tumor-infiltrating T lymphocytes (TILs) in melanoma, inducing superior immune responses [19]. Amino acids provide fuels for biosynthesis of proteins and nucleotides to promote rapid cellular growth. Given the metabolic significance of amino acids, several types of cells can take use of them to play an immunosuppressive role. Tumor-associated macrophages (TAMs), myeloid-derived suppressor cells (MDSCs), and immunotolerant dendritic cells (DCs) inhibit TILs through inducing the expression of catabolic enzymes to produce essential amino acids, such as ARG1 and indoleamine 2, 3-dioxygenase (IDO) [20–23]. For example, MDSCs inhibit anti-tumor immune responses through depleting cysteine, which is critical to T cells [24]. In conclusion, metabolism regulates the activities of both tumor cells and immunocytes, and therefore targeting metabolism may bring in therapeutic benefits to cancer patients.

## Metabolic disturbance of immune cells in the TME

### The adaptive immune cells

Accumulating evidences have demonstrated that T cells in the TME, especially Tregs, exhibit a unique metabolic phenotype [25]. Pacella and colleagues demonstrated that in murine tumor models, tumor-infiltrating Tregs accumulate intracellular lipids due to an elevated fatty acid synthesis rate [26]. As comparative advantage in glucose uptake may trigger fatty acid synthesis in intratumoral Tregs, they showed that both glycolytic and oxidative metabolism lead to expansion of Tregs. Glucose metabolism is also related with TLR8-mediated CD4^+^ Tregs immunosuppressive function inhibition in the ovarian cancer [27]. When TLR8 becomes activated, eight genes and five proteins associated with glucose metabolism in CD4^+^ Tregs are down-regulated, moreover, glucose uptake and glycolysis are also decreased. Apart from that, Weinberg et al. showed that mitochondrial respiratory chain complex III is indispensable to the immunosuppressive function of Tregs [28]. Treg-specific deletion of mitochondrial complex III causes progression of fatal inflammatory disease early in life in murine models, without influencing the cellular number of Tregs. Moreover, mice lacking mitochondrial complex III specifically in Tregs exhibit loss of T cell-suppression capacity due to increased DNA methylation as well as elevated levels of pertinent metabolites including 2-hydroxyglutarate (2-HG) and succinate, which inhibit the ten-eleven translocations (TET) family of DNA demethylases. In addition, fatty acid binding protein 5 (FABP5) affects Treg function through regulating mitochondrial integrity [29]. When inhibiting FABP5 in Tregs, oxidative phosphorylation (OXPHOS) and lipid metabolism are impaired, and the generation of cardiolipin, which is essential to mitochondrial integrity, is reduced. Subsequently, damaged mitochondria release mitochondrial DNA into the cytoplasm and trigger cyclic GMP-AMP synthase-stimulator of interferon genes (cGAS-STING)-dependent type I IFN signaling, enhancing the production of regulatory cytokine IL-10 and promoting Treg immunosuppressive activity. Tregs are not only affected by glycolysis, fatty acid metabolism and mitochondria but also acid metabolism. Serine promotes glutathione (GSH) synthesis and feeds into the one-carbon metabolic network (1CMet), which is a crucial process to Teff responses. To investigate the influence of serine metabolism in Tregs, Kurniawan et al. specifically deleted the catalytic subunit of glutamate cysteine ligase (Gclc) in Tregs in mice, inducing Treg-specific GSH loss [30]. They found that GSH-deficient Tregs show elevated serine metabolism and decreased FoxP3 expression. Furthermore, Treg-specific Gclc-deficient mice demonstrate enhanced anti-tumor responses. When serine supply is inhibited through feeding the mice with serine-deficient food, Gclc-deficient Tregs regain FOXP3 expression and immunosuppressive ability.

**Fig. 1:**
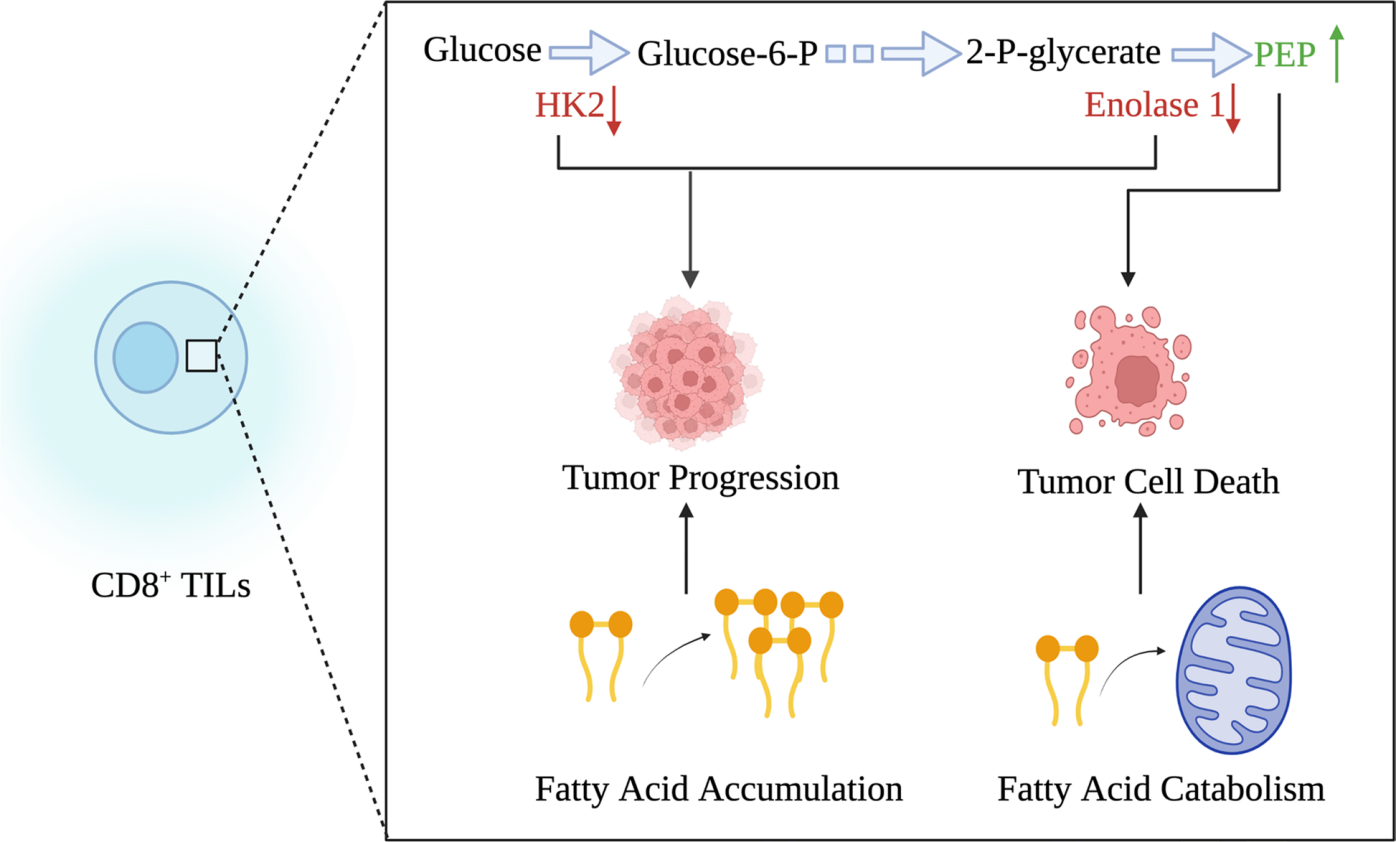
The affection of metabolic alterations on anti-tumor functions of CD8^+^ TILs. Metabolic alterations of CD8^+^ TILs mainly reflect in glycolysis and fatty acid metabolism. Reduced activity of enolase 1 and HK2 inhibits functions of CD8^+^ TILs through impairing glycolysis, whereas increased production of PEP promotes it. Compared with positive influence of fatty acid catabolism, fatty acid accumulation suppresses functions of CD8^+^ TILs. Components in Fig. 1 are drawn using tools in Biorender.com

CD8^+^ T cells engage in the anti-tumor responses and inhibit tumor progression. In the TME, metabolic alteration modulates CD8^+^ T cell function. From the aspect of glycolysis, tumor cells exhibit a preferential uptake of glucose and therefore restrict glucose supply to anti-tumor T cells [31]. For example, ovarian cancer overexpresses microRNAs miR-101 and miR-26a which limit the expression of the methyltransferase enhancer of zeste homolog 2 (EZH2), reducing glucose supply to T cells [32]. Aerobic glycolysis is critical to the formation of anti-tumor immune responses. NF-κB inducing kinase (NIK) can regulate the stabilization of hexokinase 2 (HK2) which is a key glycolytic enzyme [33]. NIK deletion induces low level of HK2 and impairs glycolysis, inhibiting the effector functions of CD8^+^ T cells in the TME (Fig. 1) [33]. In human and murine melanomas, Gemta et al. found that enolase 1, a critical glycolytic enzyme, down-regulates its activity in CD8^+^ TILs and induces defects in glycolytic metabolism of CD8^+^ TILs (Fig. 1). In addition, the downstream product of enolase 1, pyruvate, can promote glycolysis and improve the effector function of CD8^+^ TILs [34]. Besides glycolytic enzymes, the glycolytic metabolites can also affect T cell function in the TME. For example, phosphoenolpyruvate (PEP) inhibits sarco/endoplasmic reticulum Ca^2+^-ATPase (SERCA) activity to preserve the effector function of TILs. In the TME, CD8^+^ T cells up-regulate phosphoenolpyruvate carboxykinase 1 (PCK1) to promote the production of PEP, which reprograms metabolism and improves effector function (Fig. 1). The survival of melanoma-bearing mice is prolonged through overexpressing PCK1 in anti-tumor T cells [35]. Apart from glycolysis, fatty acid metabolism also has an important influence on T cells. In the hypoglycemic and anoxic TME areas of mouse melanoma models, CD8^+^ TILs strengthen peroxisome proliferator-activated receptor (PPAR)-α signaling and fatty acid catabolism, maintaining the effector function of CD8^+^ TILs. Stimulating fatty acid catabolism of CD8^+^ TILs can improve immunotherapeutic effect of melanoma (Fig. 1) [36]. In the lipid-abundant TME areas of pancreatic ductal adenocarcinoma, CD8^+^ T cells down-regulate the expression of very-long-chain acyl-CoA dehydrogenase (VLCAD) and promote the accumulation of long-chain fatty acids (LCFAs), finally impairing mitochondrial function and inducing cellular dysfunction (Fig. 1) [37]. From the perspective of amino acids, glutamine is becoming a targetable metabolite in tumor therapy [38]. In the murine triple-negative breast cancer (TNBC) model, Teffs and tumor cells compete for glutamine from the microenvironment. Tumor cells-specific deletion of glutaminase, a critical enzyme for glutamine metabolism, promotes T cell activation and enhances antineoplastic immune responses [39]. In addition, V-9302, the inhibitor against glutamine transporter, can specifically inhibit the update of glutamine in TNBC cells and but does not affect anti-tumor T cells, providing a promising therapeutic strategy for TNBC [39]. Therefore, glutamine antagonism brings in a differential therapeutic effect on tumor cells and T cells. In tumor-bearing mice, glutamine antagonism inhibits glycolytic and oxidative metabolism in cancer cells, up-regulates oxidative metabolism and induce a long-lived and highly activated phenotype in anti-tumor T cells. The differential metabolic response to glutamine antagonism between T cells and tumor cells is used as a metabolic checkpoint for tumor immunotherapy [40]. Mitochondrial morphology and metabolism are also critical to T cells. The anoxic TME promotes mitochondrial fragmentation and decreases ATP production through MYC-regulated pathways to induce T cell exhaustion [41]. Elevated mitochondrial mass represents superior respiratory capacity which is critical for generating long-lived CD8^+^ memory T cells [18, 42]. In line with this phenomena, the capacity of Teffs to generate memory T cells is impaired when deleting the mitochondrial membrane fusion protein optic atrophy 1 (OPA1) in mice [16]. In conclusion, enhancing mitochondrial fusion can prolong the persistence of CD8^+^ T cells and augment their effector function through an adoptive cell therapy [16].

In tumor tissues, the balance between antibody-producing B cells and regulatory B cells (Bregs) contribute to the patient prognosis [43]. Bregs have immunosuppressive functions, which assist tumor cells to escape immune surveillance [44]. Therefore, it is of great significance to study the influence of metabolism on the phenotypic transformation of B cells. Several studies have shown that increased glycolytic activity can induce the activation of Bregs. For example, Meng et al. found that hypoxia and hypoxia-inducible factor 1α (HIF-1α) maintain the function of Bregs by promoting glycolysis [45]. This suggests that hypoxia in the TME may induce the activation of Bregs. Ishigami et al. found that the infiltration of Bregs closely correlated to the poor prognosis in breast cancer [46]. In conclusion, the TME may induce B cells to differentiate into phenotypes favorable for tumor progression through metabolic reprogramming. However, the research for metabolism from antibody-producing B cells in the TME is still insufficient and needs further improvement.

### Innate immune cells

DCs can process and present foreign antigens to antigens-specific T cells to elicit T cells-mediated immune response. Therefore, exploring the metabolism of DCs has therapeutic implications for cancer treatment. Fatty acid synthesis has three main functional roles in DCs. First, fatty acids synthesis-induced lipid accumulation directly regulates the function of DCs. For example, lipid accumulation reduces the capacity of DCs to present antigens and activate T cells in tumor-bearing mice. T cells primed by DCs with high level of intracellular lipids show reduced capacity of proliferation [47]. Accordingly, Cubillos-Ruiz et al. found that the endoplasmic reticulum (ER) stress response factor X-box-binding protein 1 (XBP1) induces triglyceride biosynthesis and abnormal lipid accumulation in DCs and inhibit DCs to activate anti-tumor T cells. When XBP1 is deleted, DCs regain their function to stimulate T cells and induce type 1 anti-tumor immune responses [48]. Second, fatty acid synthesis can modulate the consumption of acetyl-CoA. At the steady-state, tuberous sclerosis complex subunit 1 (TSC1)- mTOR inhibit fatty acid synthesis and promote the production of acetyl-CoA in DCs to increase acetylation through down-regulating ACC1 expression [49]. Histone acetylation induced by acetyl-CoA is associated with major histocompatibility complex (MHC)-I and IL-7 genes expression. When inhibiting TSC1 in DCs, fatty acid synthesis is elevated and the level of acetyl-CoA for histone acetylation is decreased, impairing CD8^+^ T cells activation that is essential to resisting B16 melanomas. This mechanism suggests that metabolism is tightly linked to epigenetic modification [49]. Third, fatty acids synthesis expands ER and Golgi networks to promote the secretion of effector cytokines in activated DCs [50]. In addition, the mitochondrial metabolism is also directly linked to DCs. For example, deficiency of the Hippo pathway kinases Mst1/2 induces disorganized mitochondrial cristae, which then inhibit OXPHOS and impair the capacity of presenting antigens and priming CD8^+^ T cells in CD8α^+^ DCs [51].

TAMs promote tumor progression through inhibiting immunological surveillance [52]. Immunosuppressive functions of TAMs can be regulated through reprograming metabolism. For example, Wu et al. found that oleate, a type of long-chain fatty acid, relies on the mTOR pathway to induce TAM polarization and promote tumor progression [52]. Therefore, chemical inhibitors targeting the metabolism of long-chain fatty acids in TAMs provides a new therapeutic direction in cancer treatment [52]. In addition, the mitochondrial morphology of TAMs regulates the anti-tumor immunity. FAM73b, a mitochondrial outer membrane protein, is crucial to the morphological transformation of mitochondria [53]. When FAM73b is deleted, mitochondrial morphology is converted from fusion to fission in TAMs, and this conversion promotes T cell activation and enhances anti-tumor immunity. This feature highlights the therapeutic potential of targeting the molecular mechanism associated with the transformation of mitochondrial morphology [53]. TAM function is not only affected by intracellular metabolites but also extracellular metabolites. At high concentration in the TME, lactate inhibits the expression of the macrophage-specific vacuolar ATPase subunit ATP6V0d2. Consistently, ATP6V0d2-deleted macrophages show increased HIF-2α activity to mediate tumor progression. The lactate/ATP6V0d2/HIF-2α axis suggests the connection between the tumor environment and immune cells [54]. TAMs and tumor cells exert reciprocal influence on the metabolism of each other to regulate tumor progression. TAMs regulates tumor metabolism to maintain an immunosuppressive microenvironment. For example, the extracellular vesicles derived from TAMs deliver HIF-1α-stabilizing long noncoding RNA (HISLA) to promote the aerobic glycolysis and the anti-apoptotic ability in breast cancer cells. Lactate derived from aerobic glycolysis of tumor cells up-regulates HISLA in TAMs and forms a feedback loop between tumor cells and immune cells [55]. In addition, polarized M2 macrophages produce IL-6 to promote the phosphorylation of the threonine 243 of phosphoglycerate kinase 1 (PGK1^T243^) mediated by 3-phosphoinositide-dependent protein kinase 1 (PDPK1) in tumor cells, promoting glycolysis and tumor progression. Inhibiting IL-6 derived from macrophages reverses tumor progression [56]. Similarly, tumor cells can regulate macrophages metabolism to benefit their own growth. For example, peritoneal tumors, such as B16 melanoma or ID8 ovarian carcinoma, regulate the metabolism of peritoneal tissue-resident macrophages (pResMϕ), promoting OXPHOS and glycolysis and increasing itaconic acid production in pResMϕ, which lead to accelerated tumor growth. Targeting immune-responsive gene 1, which mediates production of itaconic acid in pResMϕ, proves to be a novel therapeutic strategy for peritoneal tumors [57].

MDSCs engage in inducing resistance to immune checkpoint blockade (ICB) [58]. The granulocyte macrophage-colony stimulating factor (GM-CSF) derived from tumor cells can activate STAT3 signaling pathway to induce the expression of fatty acid transport protein 2 (FATP2), which promotes lipids accumulation and enhances the immunosuppressive function in MDSCs. Inhibiting FATP2 reduces lipids accumulation and reactive oxygen species (ROS) production, inhibiting immunosuppressive functions of MDSCs and improving anti-programmed death-ligand 1 (PD-L1) tumor immunotherapy effect [58]. In the TME, MDSCs reprogram metabolic pattern from glycolysis to fatty acid oxidative metabolism and OXPHOS. Fatty acid oxidative metabolism is associated with the maintenance of immunosuppressive function of MDSCs and resistance to ICB therapy. The increased intake of exogenous fatty acid in MDSCs promotes tumor growth [59]. The serine/threonine kinase PIM1 regulates fatty acid oxidative metabolism through PPAR-γ. Inhibiting PIM1 with AZD1208 impairs MDSCs-mediated immunosuppression and improves CD8^+^ T cell-mediated antineoplastic immune response, enhancing the therapeutic effect in ICB-resistant patients [60]. Although fatty acid metabolism is important for MDSCs during tumor progression, glycolysis is found important in maintaining the survival of MDSCs in the TME. After encountering with tumor-derived factors, MDSCs up-regulate the expression of genes associated with glycolysis and increase the production of glycolytic metabolite PEP, which prevents the overproduction of ROS and promotes the survival of MDSCs. Therefore, analogs of glycolytic intermediates can be used to target MDSCs and reverse the immunosuppressive TME [61].

**Fig. 2:**
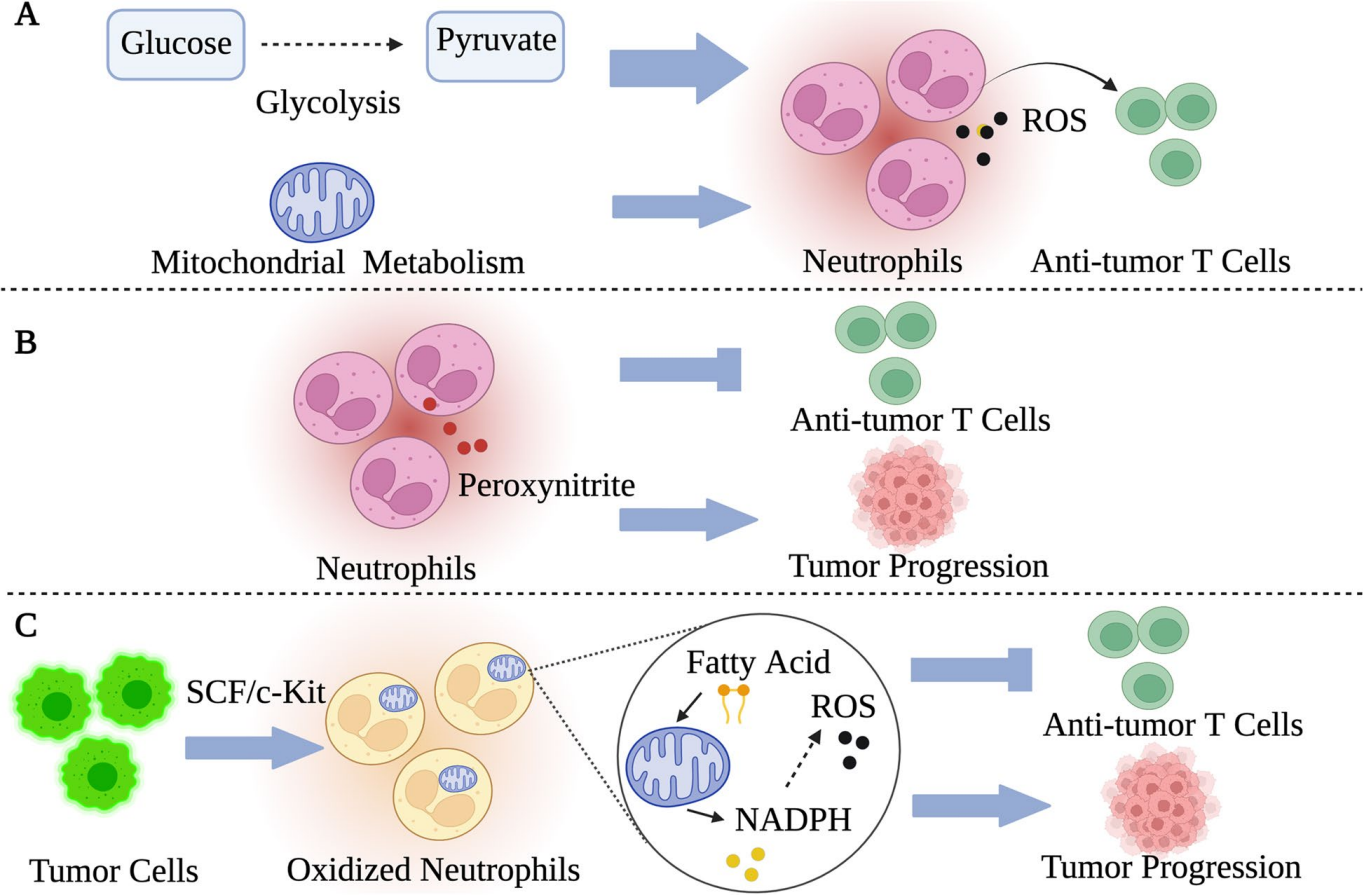
The affection of metabolic alterations of neutrophils on anti-tumor immune cells. In the past, glycolysis was considered as the main metabolism supporting immunologic function in neutrophils, while mitochondrial metabolism was less relevant to immunologic function. Recently, it is found that tumor cells can induce oxidized phenotype of neutrophils through the SCF/c-Kit signaling. Oxidized neutrophils undergo metabolic reprogramming and depend on fatty acid metabolism from mitochondria to increase ROS level and promote tumor progression. Tumor-associated neutrophils also express iNOS to induce the production of peroxynitrite, which damages anti-tumor T cells. Components in Fig. 2 are drawn using tools from Biorender.com

In cancer tissues, neutrophils can produce ROS, thus inhibiting the function of anti-tumor T cells and promoting tumor progression [62]. In the past, it was generally believed that neutrophils highly depend on glycolytic metabolism to exert immunologic function, and their functions were almost independent of mitochondrial metabolism (Fig. 2) [63]. However, Rice et al. recently found that tumors could induce neutrophils to produce oxidative phenotype through the stem cell factor (SCF)/c-Kit signaling, and the mitochondrial function of neutrophils with oxidative phenotype was enhanced (Fig. 2) [64]. Therefore, even in the TME lacking glucose, oxidized neutrophils can still maintain NADPH production through fatty acid metabolism in mitochondria to support ROS levels (Fig. 2). To sum up, tumor-induced oxidative neutrophils can overcome glucose deficiency and maintain ROS levels required by tumor immunosuppression through mitochondrial metabolism. In addition, neutrophils can also modulate anti-tumor immunity through expressing metabolic enzymes [65]. For example, in the TME, tumor-associated neutrophils (TANs) can express inducible nitric oxide synthase (iNOS) to induce the production of nitric oxide, which is further converted into peroxynitrite to damage anti-tumor T cells (Fig. 2) [66]. Neutrophil metabolism has strong potential to become the focus of future research on cancer treatment.

Natural killer cells (NK cells) can mediate the killing of cancer cells through perforin and granzymes [67]. mTORC1 is a key factor in regulating NK cell metabolism. When NK cells are activated, mTORC1 up-regulates glycolysis and OXPHOS to mediate their anti-tumor immune functions [68]. Low level of glucose in the TME impairs mTORC1-induced glycolysis and inhibits the production of IFN-γ and granzymes in NK cells [69]. Poznanski et al. isolated tumor-associated NK (taNK) cells from the TME of ovarian cancer and compared them with peripheral blood NK (pbNK) cells [70]. Relative to pbNK cells, the expression of the GLUT1 glucose transporter on taNK cells was lower, and basal and maximal OXPHOS were also significantly reduced [70]. Then, they cultivated pbNK cells in the ascites-TME (asc-TME) ex vivo and found that pbNK cells showed metabolic disturbance similar to taNK cells. CD98 is essential to glycolysis through exchanging glutamine to uptake other amino acids [71]. The cell-surface expression of CD98 in the asc-TME-incubated pbNK cells was significantly reduced [70]. Furthermore, asc-TME-incubated pbNK cells showed increased expression of proteins associated with oxidative damage and lipid peroxidation. When treating pbNK cells with RTA-408, a compound that activates antioxidant activity [72], OXPHOS and glycolysis were up-regulated and anti-tumor ability was restored [70]. Therefore, oxidative damage may be the key mechanism of TME-induced metabolic disorder in NK cells, suggesting a new scheme for cancer treatment. High lactate level in the TME can lead to increased lactate uptake by NK cells. The decrease of intracellular PH results in reduced ATP production, impaired energy potential, and dysfunction in NK cells [69, 73]. In addition, mitochondrial length of NK cells is positively correlated with granzyme levels [74]. Zheng et al. found that tumor-infiltrated NK cells up-regulated many genes related to mitochondrial fission, such as MIEF2, INF2, and FIS1 [75]. In line with it, compared with large tubular mitochondria in NK cells in normal tissues, mitochondria in NK cells in the TME are small and fragmented [75]. These results further support that NK cell dysfunction is closely related to metabolic disturbance.

## The impact of oncometabolites on the functions of immune cells

Recurrent mutations of isocitrate dehydrogenase (IDH) genes have been found in many tumors such as the lower-grade glioma (LGG), acute myeloid leukemia (AML), and cholangiocarcinoma [76]. Tumors with mutant IDH genes exhibit suppressed expression of genes related with cytotoxic T cells [77]. Mutant form of IDH proteins promotes transformation of α-ketoglutarate to the oncometabolite R-2-hydroxyglutarate (R-2-HG). R-2-HG is taken up by anti-tumor T cells and disturbs the nuclear factor transcription and polyamine biosynthesis, leading to inhibition of T cell activity [78]. Kohanbash and colleagues found that the presence of 2-HG reduces the production of the IFN-γ-inducible chemokines CXCL9 and CXCL10 through inhibiting the expression of STAT1, causing decreased infiltration of CD8^+^T cells in the TME [79]. In addition to R-2-HG, S-2-HG regulates DNA methylation to indirectly affect CD62L expression in T cells. Adoptively transferred CD8^+^ T cells treated with S-2HG ex vivo demonstrate an increased ability to proliferate and resist tumor cells in vivo, highlighting the therapeutic potential of adoptively transferred cells [80]. Several malignant cells such as AML cells with IDH mutation, can release D-2-HG, which is taken up by T cells to promote Treg frequency and inhibit the polarization of T helper cell 17 (Th17) through reprogramming metabolism towards oxidative phosphorylation [81]. In addition, lactate secreted by tumor cells exerts a direct influence on immune cells. In melanomas, increased expression of LDHA induces lactate accumulation, inhibiting tumor immunological surveillance from T cells and NK cells and leading to poor prognosis [82]. In summary, immune cells and tumor cells regulate their metabolism in a reciprocal manner.

## The metabolic fitness in anti-tumor immunity

### Metabolic adaptation in the anti-tumor function of CD8^+^ T lymphocytes

The nutrient- and oxygen-deprived TME provides a metabolic disadvantage and induces the exhaustion of tumor-infiltrating immune cells. Therefore, immune cells such as CD8^+^ T cells need to establish and maintain metabolic fitness in response to adverse metabolic conditions. When phosphatase and tensin homolog (PTEN) is accumulated on the plasma membrane through the mediation of TCR and CD28, acylglycerol kinase (AGK) positively regulates PTEN phosphorylation at the site of Ser380, Thr382, and Thr383 and inhibits the phosphatase activity of PTEN in CD8^+^ T cells, promoting activation of phosphatidylinositol-3-OH kinase (PI3K)-mTOR and mediating glycolysis and anti-tumor function. Functionally, AGK is critical to maintain metabolic fitness of CD8^+^ T cells in the TME. Deletion of AGK impairs TCR-triggered metabolic reprogramming as well as the proliferation and anti-tumor function of CD8^+^ T cells [83]. Upon TCR activation, murine CD8^+^ T cells accumulate S-2-HG which promotes the expression of TCF1, CD62L, and eomesodermin (EOMES) through regulating histone and DNA methylation, suggesting the essential connection between metabolism and epigenetics. S-2-HG treatment improves the prognosis of patients with EL4 T lymphomas overexpressing ovalbumin [80, 84]. In addition to kinases and metabolites, the mitochondrial biogenesis and architecture are associated with the effector function of CD8^+^ T cells. CD8^+^ T cells in human melanoma show decreased PPAR-γ co-activator 1α (PGC1α) which is critical to mitochondrial biosynthesis, inducing dysfunction of intratumoral T cell. PGC1α expression and mitochondrial biosynthesis maintain metabolic fitness and support nutritional needs in CD8^+^ T cells to establish sustained anti-tumor response [32, 84, 85]. Both effector CD8^+^ T cells and memory CD8^+^ T cells play important roles in cancer treatment. The transformation from effector CD8^+^ T cells into memory CD8^+^ T cells is essential to suppress tumor progression given the long-term anti-tumor immunity provided by memory CD8^+^ T cells. Inhibiting the activity of the serine/threonine kinase AKT impedes T cell survival during this transformation, suggesting that metabolic activity plays a critical role in regulating memory formation of CD8^+^ T cells [86]. In addition to AKT, PCK1 is also associated with memory CD8^+^ T cells: Ma et al. found that memory CD8^+^ T cells up-regulated PCK1 which increased glycogen production through gluconeogenesis [87]. Glycogen is subsequently catabolized into glucose-6-phosphate to initiate the pentose phosphate pathway (PPP) to produce NADPH, which is essential to increase GSH /oxidized glutathione (GSSG) ratio and inhibit ROS level in memory CD8^+^ T cells. Therefore, targeting the PCK1-glycogen-PPP axis increases ROS levels and inhibits the formation of memory CD8^+^ T cells [87]. In addition to intracellular regulation, external conditions such as diet can also affect metabolic fitness and functions of anti-tumor T cells. Rubio-Patiño et al. found that giving a low-protein diet to mice inhibits tumor progression compared with a low-carbohydrate diet [88]. A low-protein diet activates inositol-requiring enzyme 1 (IRE1α) and retinoic acid inducible gene 1 (RIG1) signaling to induce the unfolded protein response and cytokine production in cancer cells, promoting antineoplastic effector functions of CD8^+^ T cells [88]. CD8^+^ T cells with persistent metabolic fitness have demonstrated a crucial role in the optimal response to anti-cancer treatment. Besides reprogramming CD8^+^ T cells metabolism in vivo, the adoptive transfer of CD8^+^ T cells which have been cultivated ex vivo to gain metabolic fitness can also improve tumor prognosis. The culture medium with high concentration of L-arginine reduces glycolysis and elevates OXPHOS in murine T cells, inducing the acquisition of the central memory CD8^+^ T cells phenotype. Survival advantage and favorable anti-tumor effector function can be achieved when such CD8^+^ T cells are adoptively transferred into mice [84, 89].

In addition, adoptive transfer protocols of autologous T cells synergize with cholesterol biosynthesis to regulate metabolic fitness and anti-tumor functions in CD8^+^ T cells. Increased level of plasma membrane cholesterol induces enhanced TCR signaling and promotes formation of synapses. The deletion of acetyl-CoA acetyltransferase-1 (ACAT-1) increases the plasma membrane cholesterol level and improves synaptic performance in CD8^+^ T cells through up-regulating cholesterol biosynthetic enzymes. Compared with wild-type CD8^+^ T cells, the adoptive transfer of murine CD8^+^ T cells with defective ACAT-1 shows improved prognosis of melanoma tumors. Accordingly, avasimibe, an ACAT inhibitor, showed good anti-tumor effect in mouse model of melanoma [19, 84]. Hypoxia is another factor that modulates T cell function. Cytotoxic T lymphocytes (CTLs) which are adoptively transferred to treat cancer are typically cultured in 20% oxygen. Gropper et al. found that compared with 20% oxygen, CTLs which are cultured in 1% oxygen show increased release of granzyme-B and enhanced cytolysis in response to B16 melanoma cells [90].

### Metabolic rewiring of NK cells in anti-tumor immunity

**Fig. 3:**
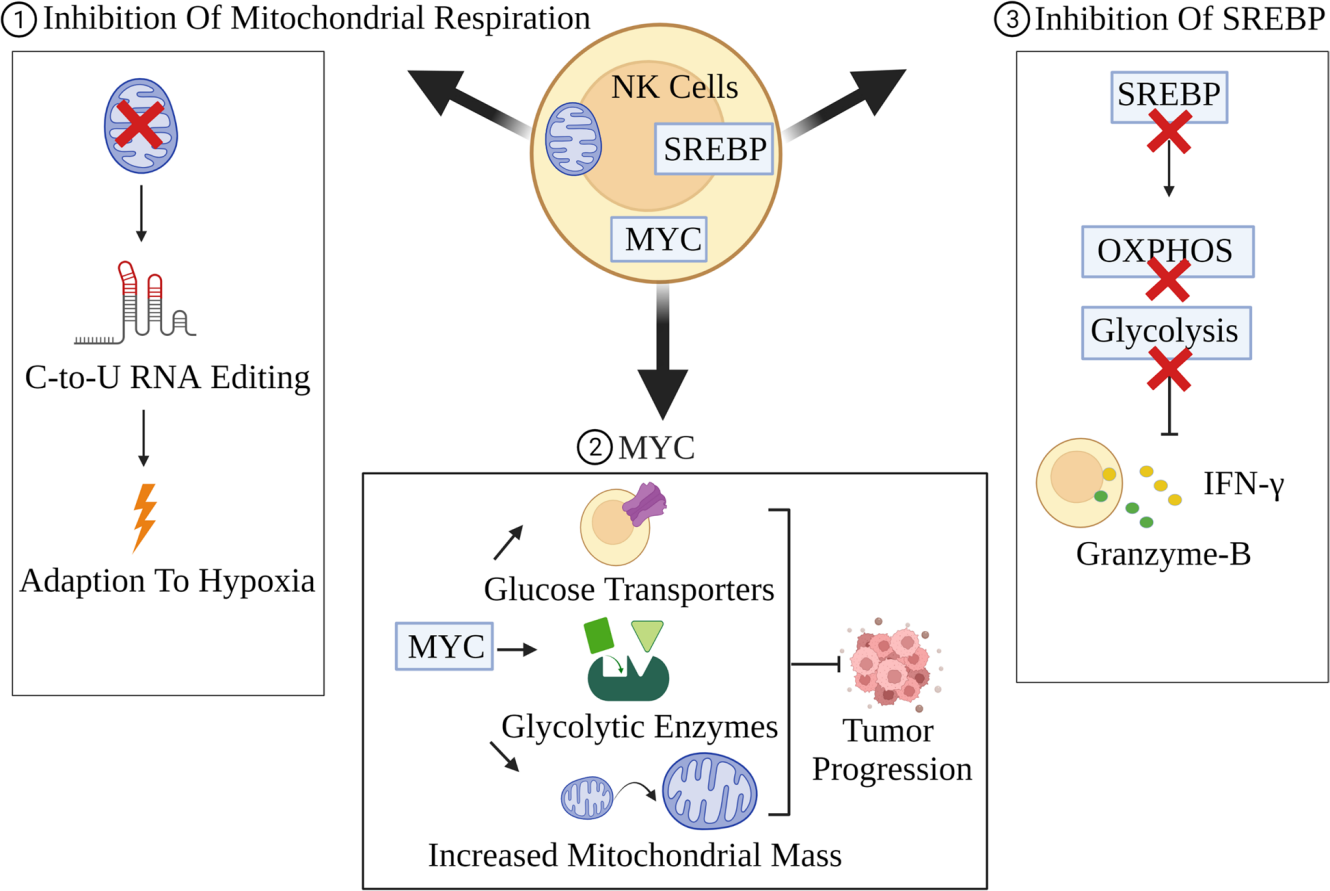
Metabolic rewiring on anti-tumor functions of NK cells**. **Metabolic rewiring is critical to maintaining anti-tumor functions of NK cells. Inhibited mitochondrial respiration induces C-to-U RNA editing in NK cells to elevate fitness in the TME with hypoxia. MYC and SREBP engage in metabolic fitness of NK cells. MYC supports OXPHOS and glycolysis in NK cells through promoting expression of glycolytic enzymes and glucose transporters and increasing mitochondrial mass, finally inhibiting tumor progression. SREBP also promotes OXPHOS and glycolysis in NK cells. Based on this, inhibiting SREBP reduces generation of IFN-γ and expression of granzyme-B, finally impairing anti-tumor cytotoxicity of NK cells. Components in Fig. 3 are drawn using tools in Biorender.com

NK cells play a key role in the immunosurveillance against tumor cells. Establishing and maintaining metabolic fitness of NK cells have therapeutic implications. Oxygen deprivation in the TME induces persistent activation of mTOR-GTPase dynamin-related protein 1 (Drp1) in NK cells, leading to mitochondrial fission to suppress cytotoxicity and survival of NK cells. Inhibiting mitochondrial fission promotes metabolic fitness and improves antineoplastic ability of NK cells [75]. In order to cope with hypoxia and improve metabolic adaptation, inhibited mitochondrial respiration induces C-to-U RNA editing in NK cells mediated by the cytidine deaminase APOBEC3G (Fig. 3) [91]. Glycolysis is associated with functional disturbance of NK cells, for example, abnormal expression of fructose-1, 6-bisphosphatase (FBP1) inhibits glycolysis and induces malfunction of NK cells. Therefore, targeting FBP1 points to a new therapeutic approach [92]. The transcription factors MYC and sterol regulatory element-binding protein (SREBP) are critical to metabolic activities of NK cells. MYC promotes glycolysis and OXPHOS through promoting expression of glucose transporters and glycolytic enzymes and providing increased mitochondrial mass (Fig. 3). In cytokines-stimulated NK cells, MYC is primarily regulated by mTORC1. Subsequently, amino acids are essential to the regulation of MYC through maintaining MYC translation and compensating MYC degradation. Instead of glutamine, the fuels for OXPHOS are provided by the citrate-malate shuttle (CMS) in NK cells. But glutamine remains critical for MYC through controlling amino acid transporter SLC7A5. Inhibiting glutaminase not only reduces glutamine consumption within tumor cells but also supports MYC without inhibiting the effector function in NK cells [71]. In addition, MYC is the downstream target of the ER stress sensor IRE1α-XBP1. The IRE1α-XBP1-MYC axis is associated with activation of NK cells [93]. In murine NK cells, SREBP promotes glycolysis and OXPHOS through regulating the expression of the citrate-malate anti-porter SLC25A1 and ACLY which are important to the CMS. When inhibiting SREBP genetically or pharmacologically, not only are glycolysis and OXPHOS inhibited, but also generation of IFN-γ and expression of granzyme-B are impaired with reduced anti-tumor cytotoxicity (Fig. 3). In human NK cells, SREBP is also associated with the generation of IFN-γ and granzyme-B. Therefore, SREBP plays an central role in metabolic reprogramming and antineoplastic effector functions of NK cells [94]. Furthermore, the short isoform of the chromatin-modifying transcriptional regulator, AT-rich interaction domain 5B (ARID5B), mediates the metabolic regulation of NK cells through facilitating mitochondrial membrane potential, genes expression associated with electron transport chain components, oxidative metabolism, and IFN-γ production [95].

### Metabolic fitness of DCs in anti-tumor immunity

DCs play an important role in tumor immune surveillance, so maintaining their metabolic fitness in the TME contributes to enhancing anti-tumor immunity. Wang et al. found that liver kinase B1 (LKB1) was an important metabolic regulator of DCs, maintaining stability of DC immune function and protecting anti-tumor immunity through inhibiting proliferation of Tregs [96]. After deleting LKB1 gene of DCs in the mouse model, mTOR in DCs was activated, and then DCs underwent metabolic disorder, finally leading to abnormal DC maturation and uncontrolled generation of immunoregulatory factors [96]. Therefore, LKB1 signaling in DCs may become a promising target of cancer therapy through regulating Treg proliferation. Hu et al. found that, in the mouse colorectal cancer model, DC-derived ROS induced interferon inducible protein 204 (IFI204) deSUMOylation through promoting accumulation of SUMO-specific protease 3 (SENP3), finally enhancing STING activation and anti-tumor immunity in DCs [97]. This study illustrates the correlation between the metabolic byproduct and immunologic function in DCs, and provides a new strategy for tumor treatment.

## Other factors implicated in immune cell metabolism

### Diet

Metabolism is closely related with diet. Alterations in diet affect metabolic activities and thus have an influence on function of immune cells and tumor progression. Under normal metabolic condition, low level of autophagy in tumor cells can induce the expression of CD39, which then increases extracellular concentration of adenosine through promoting processing of extracellular ATP into adenosine. Elevated adenosine can directly and indirectly inhibit CD8^+^ T cells through promoting Tregs [98]. Pietrocola et al. found that caloric restriction induces increased tumor cell autophagy and inhibits expression of CD39, causing the accumulation of ATP which stimulates the effector functions of CD8^+^ T cells but inhibits immunosuppressive functions of Tregs. They showed that starvation combined with chemotherapy drugs such as mitoxantrone or oxaliplatin inhibit tumor cell growth in multiple models including fibrosarcoma and colorectal cancer [99]. Under normal nutritional supply, heme-oxygenase1 (HO-1) acts to inhibit apoptosis [98]. Di Biase et al. showed that caloric restriction reduces the expression of HO-1, inducing apoptosis in tumor cells and inhibiting Tregs. Apoptotic tumor cells stimulate CD8^+^ T cells which further mediate tumor cell apoptosis through a positive feedback loop [100]. Fasting-mimicking diet (FMD) synergizes chemotherapy drugs doxorubicin or cyclophosphamide to inhibit tumor cells growth. FMD can also increase the number of CD8^+^ T cells and prevent them from chemotherapy-induced cell death [100]. Vernieri et al. studied the metabolic and immunological alterations in 101 cancer patients after an FMD regimen was administered to these patients [101]. They found that FMD could reshape metabolism and thus alter anti-tumor immunity. The following mechanisms were proposed to explain this phenomenon. Carbohydrate restriction induced low levels of GM-CSF, CCL2, and G-CSF, impairing migration of immunosuppressive monocytes [101, 102]. In addition, carbohydrate restriction also induced high levels of ketone body in blood and thus reduced expression of PD-L1 in monocytes, inhibiting immunosuppressive function of monocytes [101, 103]. Protein restriction reduced insulin-like growth factor 1 (IGF-1) in blood and thus inhibited the IGF-1/IGFR1/PI3K/AKT/mTORC1 axis in tumor cells, TAMs, and Tregs, activating anti-tumor T cells [101, 104, 105]. In summary, caloric restriction promotes antineoplastic immune responses and suppresses tumor cell proliferation. Many cancer therapeutics produce adverse effects on immune cells due to similar metabolic pattern between immune cells and tumor cells [106, 107]. However, caloric restriction has been shown to increase cytotoxicity and cellular count of CD8^+^ T cells, producing a win-win outcome. Therefore, caloric restriction as an adjuvant therapy with immunotherapy and chemotherapy may generate promising therapeutic efficacy.

Creatine supplementation through diet can inhibit tumor progression in B16 melanoma model and M38 colon cancer model in mice. Maintenance of the anti-tumor effector function of CD8^+^ T cells is dependent on energy production. Glycolysis or the TCA cycle can transform nutrients into energy in the form of ATP. Creatine can store redundant ATP in case of excessive need. The metabolic stress of competing for limited nutrients with tumor cells causes shortage of nutrients in CD8^+^ T cells [108]. In response to this, creatine releases stored ATP to support anti-tumor response, relieving the energy stress elicited by nutrient shortage. Creatine is therefore deemed as a “molecular battery”, storing energy which can be used by T cells to resist the metabolic stress from tumor cells [108]. In line with it, TILs up-regulate the expression of creatine transporter such as SLC6A8 and creatine transporter (CRT) [109].

### Obesity

Obesity-induced lipotoxic environment promotes the up-regulation of PPARαδ genes which drive lipid accumulation in NK cells, which are critical to anti-tumor immune response. Subsequently, up-regulated PPARαδ inhibits mTOR-mediated glycolysis, impedes the generation of IFN-γ and cytotoxic granules, and prevents the recruitment of cytotoxic granules into the synapses between NK cells and tumor cells [110]. Consistently, inhibiting PPARαδ or impeding lipid transportation into the mitochondria can restore the cytotoxicity of NK cells [110]. Obesity-induced cancer progression is associated with dysregulated inflammation which is mediated by STAT3 [111, 112]. For example, adipocytes from obese patients with breast cancer produce high levels of fatty acids and adipokines (such as leptin) to promote tumor progression [113–115]. Leptin activates the JAK2-STAT3 signaling pathway in activated T cells [116]. FAO is associated with long-term persistence of cells maintaining CD8^+^ T memory cells phenotypes [18, 117]. Glycolysis is associated with short-term persistence and high proliferation of cells maintaining CD8^+^ Teffs phenotypes. STAT3 reprogram metabolisms through promoting FAO and inhibiting glycolysis in CD8^+^ Teffs, leading to suppression of anti-tumor response [118]. STAT3-induced inhibition of glycolysis is associated with reduced secretion of IFN-γ and other T helper cell 1 (Th1) cytokines [118]. Therefore, the leptin-STAT3-FAO axis is the mediator between obesity and impaired anti-tumor immune response. In order to study the effect of obesity on anti-tumor immune responses, Ringel et al. compared the effect of high-fat diet (HFD) on the metabolism of tumor cells and T cells [119]. They found that, compared to control diet (CD), cancer cells took up more fatty acids but CD8^+^ T cells did not increase the utilization of fatty acids. This indicated that, when feeding HFD in mice, tumor cells demonstrated higher metabolic adaptability than CD8^+^ T cells and probably induced low fatty acid uptake of CD8^+^ T cells, inhibiting anti-tumor immune responses of CD8^+^ T cells. When overexpressing prolyl hydroxylase-3 (PHD3) to inhibit fatty acid uptake of tumor cells in mice, anti-tumor immune responses mediated by CD8^+^ T cells were enhanced [119].

In the anaerobic intestinal microenvironment, gut microbiota can convert polysaccharides that cannot be absorbed by the gastrointestinal tract into short-chain fatty acids (SCFAs), such as acetate, butyrate and propionate [120]. He et al. found that the gut microbial metabolite butyrate stimulates the IL-12 signaling pathway to promote CD8^+^ T cell-mediated anti-tumor response, leading to improved efficacy of the oxaliplatin therapy. Accordingly, the oxaliplatin-sensitive patients show higher level of serum butyrate as compared with that of patients with oxaliplatin-resistant tumor [121]. The intestinal flora of obese patients is often dysbiotic, and the abundance of gut microbiota producing SCFAs, especially butyrate producing-bacteria, is lower than people with normal body mass index [122]. In summary, the study on the relationship among obesity, microbial metabolism, and immune cells may become a promising field for future cancer treatment.

## The therapeutic clues offered by immunometabolic alterations for cancer patients

Inhibiting tumor progression through metabolic manipulation has been widely studied. For example, glutamine-synthetase (GS) catalyzes the conversion of glutamate to glutamine. When GS is specifically inhibited in macrophages, intracellular succinate is increased and intracellular glutamine is reduced, accompanied with elevated glycolysis and a phenotypic switch from M2 to M1 [123]. Compared with immunosuppressive M2 phenotype, M1-like macrophages induced by pharmacologic inhibition of GS recruit T cells to resist tumor cells. Macrophage-specific GS inhibition in tumor-bearing mice shows increased accumulation of cytotoxic T cells with the inhibition of metastasis. Targeting GS through genetic deletion or pharmacological inhibition provides optimism for preventing cancer metastasis [123].

Metabolism-induced dysfunction of NK cells in the TME has brought significant obstacles to cancer treatment, so the therapeutics targeting NK cell metabolism may greatly improve the efficacy of cancer treatment. STAT3 signaling plays a key role in mediating Warburg effect, which is beneficial to the survival of tumor cells in the TME with nutritional deficiency [124]. Based on this, Poznanski et al. studied a special type of NK cells, whose expansion were driven by STAT3/IL-2 signaling [70]. They found that expanded NK (exNK) cells up-regulated glycolysis and down-regulated OXPHOS, showing metabolic changes similar to Warburg effect of tumor cells. In the previous chapter, we have mentioned that pbNK cells in the asc-TME show oxidative damage, which induces disordered metabolism and impaired anti-tumor immune function. Poznanski et al. compared exNK cells with pbNK cells from the perspective of metabolism and function [70]. They found that exNK cells could down-regulate the expression of proteins related to oxidative damage and upregulate the expression of proteins related to DNA repair, thus resisting oxidative stress. In addition, exNK cells showed flexible utilization of metabolic substrates through expressing enzymes associated with one-carbon metabolism, folate metabolism, serine synthesis pathways. Compared with pbNK cells, exNK cells in the asc-TME showed a stronger ability to kill cancer cells. exNK cells with metabolic flexibility and adaptability can better adapt to the TME and exhibit enhanced anti-tumor ability, so they may have a broader impact in the field of cancer treatment.

Liu et al. found that Tregs and tumor cells in the TME could induce lipid metabolism alteration of T cells through promoting the expression of group IVA phospholipase A and eventually induce T cell senescence [125]. Senescent T cells showed reduced ability to kill tumor cells. After inhibiting group IVA phospholipase A in T cells in mouse models with breast cancer and melanoma, they found that lipid metabolism of T cells was reprogrammed and anti-tumor ability of T cells was enhanced [125]. Therefore, targeting lipid metabolism in T cells is probably synergistic to enhance the therapeutic effect of cancer therapy. Compared to other tissues, Tregs in cancer tissues show high expression of CD36, which can promote adaptation of Treg in the TME with high lactate levels through peroxisome proliferator-activated receptor-β signaling [126]. Mouse models with genetic knockout of CD36 in Tregs show reduced tumor-infiltrated Tregs and increased anti-tumor T cells [126]. Therefore, targeting CD36 in Tregs probably inhibits metabolic fitness of Tregs in the TME and improves tumor prognosis. In addition to Tregs, CD36 also affects the metabolism and function of CD8^+^ T cells. CD36 expressed on the CD8^+^ T cell surface promotes oxidized low-density lipoproteins (OxLDL) uptake by CD8^+^ T cells, inducing lipid peroxidation and activating downstream P38 kinase [127]. Xu et al. found that dysfunction of CD8^+^ TILs was recovered when reducing lipid peroxidation through overexpressing glutathione peroxidase 4 [127]. This suggests that therapy targeting the CD36/ lipid peroxidation axis has the potential to enhance anti-tumor immunity. Bian et al. found that tumor consumes methionine far more than T cells through higher expression of the methionine transporter SLC43A2 [128]. The low level of methionine in T cells led to loss of dimethylation at lysine 79 of histone H3 (H3K79me2) and low expression of STAT5, thus inhibiting the immune function of T cells. After supplementing methionine in tumor-bearing mice or inhibiting SLC43A2 expression in tumor cells, H3K79me2 and STAT5 expression were elevated and anti-tumor immune response of T cells was elevated [128].

## Adjuvant therapy informed by metabolic reprogramming for other cancer therapies

Targeting metabolism gradually becomes an effective option of adjuvant therapy to numerous promising anti-cancer therapies including (I) adoptive transfer protocols of autologous T cells; (II) oncolytic viruses; and (III) immune checkpoint blockade. Metabolic manipulation can combine with these approaches to augment the anti-tumor immune response.

In adoptive transfer protocols of autologous T cells, the effect of metabolic manipulation during in vitro culturing has been reported [12]. For example, glycolysis of T cells cultured in vitro can be inhibited through blocking hexokinase with the glucose analogue 2-deoxyglucose (2-DG). This treatment promotes the generation of T cells possessing memory phenotype associated with improved survival and superior anti-tumor effector function (Table 1) [129]. Memory-associated metabolism (such as FAO and OXPHOS) of T cells cultured in vitro can be augmented through the use of small-molecule inhibitors that promote mitochondrial fusion and inhibit mitochondrial fission. Such treatment generates CD8^+^ T cells with superior anti-tumor function [16].

**Table 1:** The synergy between targeting metabolism and existing anti-cancer therapies

Existing Anti-cancer Therapies	Metabolic Manipulation	Purpose Of Manipulation	Synergistic Mechanism	Reference
Adoptive transfer protocols of autologous T cells	Glucose analog 2-DG	Inhibiting glycolysis	Generation of T cells with memory phenotype	Sukumar et al .[129]
Oncolytic viruses	Engineering oncolytic viruses to express leptin	Increasing FAO and OXPHOS and promoting mitochondrial biogenesis	Differentiation of T cells into memory-like phenotype	Rivadeneira et al .[130]
Immune checkpoint blockade	Mitochondrial activators	Activating FAO and OXPHOS and promoting mitochondrial expansion	Enhanced activation and proliferation of CTLs	Chamoto et al .[131]

Oncolytic viruses induce tumor cell lysis and initiate immune responses. However, oncolytic viruses-initiated immune response may not be sufficient due to the TME with limited nutrients. Engineering oncolytic viruses to express leptin can augment anti-tumor response. Leptin can reprogram metabolism through increasing FAO and OXPHOS, and it also promotes mitochondrial biogenesis, inducing differentiation of T cells into memory-like phenotypes which is critical to generate efficient anti-tumor effects (Table 1) [130]. Consistently, abundant T memory cell populations are observed with concordant anti-tumor response in the TME after leptin-expressing oncolytic virus therapy. Therefore, enabling oncolytic viruses to import metabolic regulators into the TME may further improve their anti-tumor response [130].

Immunotherapy with PD-1 blockade has been widely used, but there are still significant portion of patients with less optimal response to the treatment. Mitochondrial activators include ROS, uncouplers, AMPK activators, mTOR activators, and PGC1α activators. Mitochondrial uncouplers have been shown to synergize with PD-1 blockade. When the AMPK and the mTOR pathway associated with mitochondrial metabolism are activated, their downstream PGC1α and T-box expressed in T cells (T-bet) are induced to up-regulate, which activates FAO and OXPHOS and promotes mitochondrial expansion in tumor-reactive CTLs, finally enhancing activation and proliferation of CTLs (Table 1). In conclusion, the therapeutic efficacy of PD-1 blockade can be enhanced when synergizing with mitochondrial activators which recover mitochondrial metabolism. Recently, PGC1α activators bezafibrate and oltipraz have moved into clinical study [131].

The therapeutic significance of metabolism has been evaluated in several clinical trials targeting immunometabolism to improve anti-tumor response. For example, Mussai et al. found that arginase II activity in AML blasts could induce a low arginine microenvironment which was associated with T cell exhaustion. Targeting arginase II ex vivo enhanced chimeric antigen receptor T cell (CAR-T cell) proliferation and cytotoxicity to resist AML blasts. Therefore, measuring arginine concentration in the plasma and inhibiting arginase have a good prospect to enhance the effect of immunotherapy to treat AML blasts [132]. In addition, a large number of patients with metastatic melanoma show no or minimal responses to ICB therapy. Mussai et al. reported that defective expression of ornithine transcarbamylase (OTC) and argininosuccinate synthetase (ASS) induces arginine-auxotrophic tumor cells and causes these tumor cells to depend on extracellular arginine [133]. In a phase I study (NCT02285101), De Santo et al. first reported that an arginine-auxotrophic melanoma patient with resistance to ICB therapy was treated with pegylated recombinant arginase BCT-100 to induce systemic arginine depletion and exhibited tumor remission with the duration of 30 months [134]. This case shows that ICB therapy may be combined with BCT-100 metabolic therapy to improve the curative effect for arginine-auxotrophic melanoma in the future.

Targeting metabolism in cancer cells can not only affect the malignant population but also immune cells. Two major pathways have been implicated to prevent this phenomenon. The first pathway is to augment immune cells with not promoting survival and growth of tumor cells. The second pathway is to inhibit metabolisms which cancer cells are addicted to but immune cells show little dependence on [12]. The mechanism of the second pathway is associated with the relative metabolic inflexibility of tumor cells, as compared to immune cells. For example, tumor cells show strict dependence on glycolysis, but T cells can sustain glucose deprivation through differentiating into longer-lasting anti-tumor memory T cells, which exhibit minimal dependence on glycolysis [129, 135].

## Conclusion and future perspective

Metabolism plays a central role in cellular survival. Immune cells display distinct metabolic characteristics as compared to malignant cells. Therefore, insights into the unique metabolic pattern of immune cells allow us to enhance the immune system on the surveillance and inhibition of tumor progression. In addition, development of novel technologies enables new therapeutic opportunities to target metabolism. Application of such metabolism-targeting therapies may bring in therapeutic benefits to cancer patients, adding potential synergy with immune therapies.

## Data Availability

Not applicable.

## Abbreviations

Acetyl-CoA: Acetyl-coenzyme A
ACC1: Acetyl-CoA carboxylase 1
Acyl-CoA: Acyl-coenzyme A
ACAT1: Acyl-CoA: cholesterol transferase 1
ACAT-1: Acetyl-CoA acetyltransferase-1
AGK: Acylglycerol kinase
AMPK: AMP-activated protein kinase
AML: Acute myeloid leukemia
ARG1: Arginase 1
ARID5B: AT-rich interaction domain 5B
asc TME: Ascites-TME
ASS: Argininosuccinate synthetase
Bregs: Regulatory B cells
CAR-T cell: Chimeric antigen receptor T cell
CD: Control diet
cGAS-STING: Cyclic GMP-AMP synthase-stimulator of interferon genes
CMS: Citrate-malate shuttle
CPT1A: Carnitine palmitoyltransferase 1A
CRT: Creatine transporter
CTLs: Cytotoxic T lymphocytes
DCs: Dendritic cells
Drp1: Dynamin-related protein 1
EOMES: Eomesodermin
ER: Endoplasmic reticulum
exNK: Expanded NK
EZH2: Enhancer of zeste homolog 2
FABP5: Fatty acid binding protein 5
FATP2: Fatty acid transport protein 2
FBP1: Fructose-1, 6-bisphosphatase
FMD: Fasting-mimicking diet
Fru6P: Fructose-6-phosphate
Gclc: The catalytic subunit of glutamate cysteine ligase
GM-CSF: Granulocyte macrophage-colony stimulating factor
GSSG: Oxidized glutathione
GS: Glutamine-synthetase
GSH: Glutathione
HBP: Hexosamine biosynthetic pathway
HFD: High-fat diet
HISLA: HIF-1α-stabilizing long noncoding RNA
HIF-1α: Hypoxia-inducible factor 1α
HK2: Hexokinase 2
HO-1: Heme-oxygenase1
H3K97me2: Dimethylation at lysine 79 of histone H3
ICB: Immune checkpoint blockade
IDH: Isocitrate dehydrogenase
IDO: Indoleamine 2,3-dioxygenase
IGF-1: Insulin-like growth factor 1
IFI204: Interferon inducible protein 204
IFN-γ: Interferon-γ
IL: Interleukin
iNOS: Inducible nitric oxide synthase
IRE1α: Inositol-requiring enzyme 1
LCFAs: Long-chain fatty acids
LDH: Lactate dehydrogenase
LGG: Lower-grade glioma
LKB1: Liver kinase B1 (LKB1)
MAPK: Mitogen-activated protein kinase
MDSCs: Myeloid-derived suppressor cells
MHC: Major histocompatibility complex
mTOR: Mammalian target of rapamycin
NIK: NF-κB-inducing kinase
NK: Natural killer
OPA1: Optic atrophy 1
OTC: Ornithine transcarbamylase
OxLDL: Oxidized low-density lipoproteins
OXPHOS: Oxidative phosphorylation
pbNK: Peripheral blood NK
PCK1: Phosphoenolpyruvate carboxykinase 1
PD-L1: Programmed death-ligand 1
PDPK1: 3-phosphoinositide-dependent protein kinase 1
PEP: Phosphoenolpyruvate
PGC1α: PPAR-γ co-activator 1α
PHD3: Prolyl hydroxylase-3
PI3K: Phosphatidylinositol-3-OH kinase
PPAR: Peroxisome proliferator-activated receptor
PPP: Pentose phosphate pathway
pResMϕ: Peritoneal tissue-resident macrophages
PTEN: Phosphatase and tensin homolog
RIG1: Retinoic acid inducible gene 1
ROS: Reactive oxygen species
R-2-HG: R-2-hydroxyglutarate
SCF: Stem cell factor
SCFAs: Short-chain fatty acids
SENP3: SUMO-specific protease 3
SERCA: Sarco/endoplasmic reticulum Ca^2+^-ATPase
SREBP: Sterol regulatory element-binding protein
TAMs: Tumor-associated macrophages
TANs: Tumor-associated neutrophils
taNK: Tumor-associated NK
T-bet: T-box expressed in T cells
TCA: Tricarboxylic acid
TCR: T-cell receptor
Teff: Effector T cell
TET: Ten-eleven translocations
Tregs: Regulatory T cells
TSC1: Tuberous sclerosis complex subunit 1
Th17: T helper cell 17
Th1: T helper cell 1
TILs: Tumor-infiltrating T lymphocytes
TME: Tumor microenvironment
TNBC: Triple-negative breast cancer
UDP-GlcNAc: Uridine diphosphate N-acetylglucosamine
VLCAD: Very-long-chain acyl-CoA dehydrogenase
XBP1: X-box-binding protein 1
1CMet: One-carbon metabolic network
2-HG: 2-hydroxyglutarate
2-DG: 2-deoxyglucose
